# Familiar observers attenuate surgery‐induced neuroinflammation and cognitive dysfunction in mice

**DOI:** 10.1111/cns.14351

**Published:** 2023-07-05

**Authors:** Qun Jiang, Mingyan Guo, Zhiyi Zuo

**Affiliations:** ^1^ Department of Anesthesiology University of Virginia Charlottesville Virginia USA; ^2^ Department of Anesthesiology The Second Affiliated Hospital of Guangzhou University of Chinese Medicine Guangzhou China; ^3^ The First School of Clinical Medicine Southern Medical University Guangzhou China; ^4^ Department of Anesthesiology, Sun Yat‐sen Memorial Hospital Sun Yat‐sen University Guangzhou China

**Keywords:** anxiety, familiar observer, neural circuitry, neuroinflammation, postoperative cognitive dysfunction

## Abstract

**Aims:**

Postoperative cognitive dysfunction (POCD) is a common complication associated with poor outcome. Our previous study has shown that living with familiar observers in the same cage reduces anxiety of mice with surgery. Anxiety can impair learning and memory. Thus, this study was designed to determine whether living with familiar observers attenuated the dysfunction of learning and memory of mice with surgery.

**Methods:**

Six‐ to eight‐week‐old CD‐1 male mice or 18‐month‐old C57BL/6 male mice had left carotid artery exposure under isoflurane anesthesia. They lived with non‐surgery male mice at 2 (number of surgery mice) to 3 (number of non‐surgery mice) ratio or with other surgery mice. Mice were subjected to light and dark box test 3 days after surgery to measure their anxiety levels and novel object recognition and fear conditioning tests from 5 days after surgery to measure their learning and memory. Blood and brain were harvested for biochemical analysis.

**Results:**

Living with familiar observers that lived with surgery mice for at least 2 weeks before the surgery and then after surgery reduced the anxiety and dysfunction of learning and memory in young adult male mice. Living with unfamiliar observers that lived with surgery mice after the surgery but not before the surgery did not have those effects on the mice with surgery. Living with familiar observers attenuated learning and memory dysfunction after surgery also in old male mice. Living with familiar observers attenuated inflammatory response in the blood and brain and the activation of the lateral habenula (LHb)‐ventral tegmental area (VTA) neural circuitry, which has been shown to be important for POCD. Wound infiltration with bupivacaine attenuated the activation of LHb‐VTA.

**Conclusion:**

These results suggest that living with familiar observers attenuates POCD and neuroinflammation, possibly via inhibiting the activation of the LHb‐VTA neural circuitry.

## Graphical abstract

Living with familiar non‐surgery cage‐mates attenuated surgery‐induced anxiety, activation of the lateral habenula (LHb)‐ventral tegmental area (VTA) neural circuitry, neuroinflammation, and POCD.

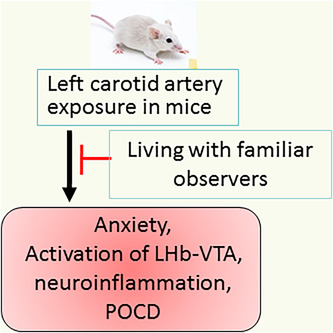

## 1 INTRODUCTION

Postoperative cognitive dysfunction (POCD) is a common complication affecting 20%–40% of patients of all ages a few days after surgery and about 10% of elderly patients a few months after surgery.^1^, ^2^ POCD is associated with poor outcome including a longer hospital stay and higher mortality.^1^, ^2^, ^3^ Thus, interventions to reduce POCD are urgently needed.

Patients with surgery often are anxious.^4^, ^5^ Anxiety can impair learning and memory.^6^ It is not known whether anxiety during the perioperative period contributes to POCD. Our previous study has shown that mice with surgery are anxious and that living with familiar observers without surgery in the same cages reduces this anxiety.^7^ Currently, it is a common practice that mice with surgery live together in a cage in animal study. Although some patients may not have family members living with them after surgery, many patients with surgery live with their family members after they are discharged from the hospital. Thus, current animal models for POCD do not fully represent clinical scenarios. The effects of living with non‐surgery subjects on the development of POCD are not known.

Neuroinflammation is considered the fundamental pathology for POCD.^8^, ^9^ Our recent study has shown that surgery activates the lateral habenula (LHb)‐ventral tegmental area (VTA) neural circuitry, which induces a series of changes including neuroinflammation in the brain to lead to POCD.^10^ Interestingly, reducing nerve input from the surgical wound by injecting local anesthetic to the wound area inhibits neuroinflammation and the development of POCD.^11^ It is not known whether wound infiltration with a local anesthetic blocks the activation of the LHb‐VTA neural circuitry.

Based on the above information, we hypothesize that living with familiar observers without surgery reduces anxiety, neuroinflammation, and activation of LHb‐VTA and POCD in mice with surgery. To address this hypothesis, mice with surgery lived with other surgery mice or non‐surgery mice after surgery. Their anxiety, learning, memory, and neuroinflammation were evaluated.

## 2 METHODS AND MATERIALS

The protocol of animal experiments in this study was approved by the Institutional Animal Care and Use Committee at the University of Virginia (Charlottesville, VA, USA). All animal experiments were conducted in accordance with the National Institutes of Health Guide for Care and Use of Laboratory Animals (NIH publication number 80–23, revised in 2011) and reported according to the ARRIVE guidelines.

### 2.1 Animals and animal groups

Six‐ to eight‐week‐old CD‐1 male mice (weighing 31–36 g, young adult mice) from Charles River Laboratory Inc. were maintained under a 12 h light/dark cycle with free access to food and water. All experimental procedures and behavior tests were conducted during the light phase.

In the first experiment, the mice were randomized by a SPSS‐generated random number assignment to one of the three groups: control, surgery, and surgery with familiar observer. Mice lived together for 2 weeks before surgery (5 mice per cage that was 26 × 15 × 12 cm). These five mice in a cage were cage‐mates throughout the experiments. Control mice lived together with other control mice. Mice with surgery in the surgery group lived together with other mice with surgery before and after the surgery. Only two of five mice per cage in the surgery with familiar observer group underwent surgery and were placed back to the same cage with the other three familiar non‐surgery cage‐mates. These mice were used for behavioral tests (dark and light box, novel objection recognition, and fear conditioning). The second cohort of mice, also in three groups, was used to obtain the serum and hippocampus for ELISA analyses of interleukin (IL)‐6 and corticosterone at 24 h after surgery. Half of the brain of these mice was harvested for allograft inflammatory factor 1 (Iba‐1) immunofluorescence staining. The third cohort of the same three groups was used to harvest the brain at 3 h after surgery. These brains were used for c‐Fos immunofluorescence staining in the LHb and VTA.

In the second experiment, mice were randomly assigned to two groups: surgery and surgery with unfamiliar observer. Mice in the surgery group were treated in the same way as stated in the first experiment. Two of five mice per cage in the surgery with unfamiliar observer group underwent surgery and were placed to a different cage with three unfamiliar no‐surgery mice. These mice were used for behavioral tests.

In the third experiment, mice were randomized to two groups: surgery and surgery with bupivacaine group. Mice in the surgery group had the same treatment as stated in the first experiment. Mice in the surgery with bupivacaine group had surgery and received infiltration of 0.25% bupivacaine to the surgical site before skin incision was made. Their brains were harvested at 3 h after surgery for c‐Fos immunofluorescence staining in the LHb and VTA.

In the fourth experiment, 18‐month‐old male C57BL/6 mice weighing 26–38 g (old mice) from the National Institute on Aging (Bethesda, MD, USA) were randomly assigned to four groups: control, surgery, surgery with familiar observer, and familiar observer. The first three groups were treated in the same way as described for the first experiment except that each cage had four mice. Thus, two mice with surgery lived with two non‐surgery mice in the surgery with familiar observer group. The mice in the familiar observer group were those from the surgery with familiar observer group. These mice were used for behavioral tests (dark and light box and fear conditioning).

### 2.2 Animal surgery

The left carotid artery exposure surgery was used as the model to induce POCD.^12^ Briefly, mice were anesthetized by 1.8% isoflurane whose concentration was monitored with a DatexTM infrared analyzer (Capnomac, Helsinki, Finland). Their temperature was maintained by a warm pad. The mouse was placed in a supine position and a 2‐cm midline neck incision was made after mouse was exposed to isoflurane at least for 30 min. The soft tissues over the trachea were retracted gently. One‐centimeter long left common carotid artery was dissected carefully free from adjacent tissues without damaging the vagus nerve. The wound was then irrigated and closed by using surgical sutures. The surgical procedure was performed under sterile conditions and lasted about 15 min. The total duration of anesthesia was 2 h, a clinically relevant duration of anesthesia. No response to foot pinching was observed during the whole course of anesthesia. During anesthesia, rectal temperature was monitored and maintained at 37°C with the aid of servo‐controlled warming blanket (TCAT‐2LV, Physitemp instruments, Clifton, NJ).

### 2.3 Light and dark box test

Mice were subjected to this test 3 days after surgery. The light and dark box was made of white and black opaque Plexiglas (19 cm in width × 19 cm in length × 25 cm in height) to form light chamber and dark chamber. These two chambers were connected by a central gray corridor (6.5 cm in width × 9 cm in length × 25 cm in height). Mice were placed in the middle of the light chamber facing a side away from the door and then released. Behavior was recorded for 5 min. The time spent in each chamber and the crossing times through the corridor were recorded. After each trial, the apparatus was cleaned with 70% ethanol.

### 2.4 Novel object recognition test

This test was performed 5 days after surgery. As we had described before,^13^ mice were put in an open field chamber for 5 min for habituation on the fourth day after surgery. The test was performed in the following day. Two of the same objects were placed at adjacent angles of the chamber on the learning day. Mice were put into the chamber with their backs turned toward the objects and allowed to explore the chamber freely for 5 min. One of the objects was replaced by a novel object 24 h later. Mice were put into the chamber with their backs turned toward the objects and allowed to explore for 5 min. Animal behavior was recorded by ANY‐maze behavioral tracking software (Stoelting Co). Exploratory time of new (T2) and old (T1) objects within 5 min was recorded. The memorization ability of the mouse was quantified by discrimination index (DI): DI = T2/(T1 + T2). The field was always provided with even light, and the objects and fields were cleaned with 70% ethanol after each trial.

### 2.5 Fear conditioning test

Twenty days after the surgery, the mouse was subjected to fear conditioning test using the Freeze Monitor (Instruments, San Diego, CA) in the same way as we described before.^12^, ^14^ Briefly, three phases were included: the training, the context‐related memory and the tone‐related memory. During the training phase, mice were placed in a test chamber and received a three tone‐foot shock pairings (tone: 2000 Hz, 85 db, 30 s; shock: 0.7 mA, 2 s) with a 1 min interval in a relatively dark room. The chamber was cleaned with 70% ethanol after each trial. In the context‐related memory test, the mouse was placed back to the same chamber 24 h later for 6 min in the absence of tone and shock. The duration of freezing behavior was recorded in this 6 min. In the tone‐related memory test, the mouse was placed 2 h later in a new test chamber that had a different context and smell environment (this chamber was wiped with 1% acetic acid) for 7.5 min in a relatively bright room. After 3 min acclimatization time, the 30 s tone stimulus with 1 min interval was turned on for 3 cycles that lasted for 4.5 min in total. The freezing behavior in this 4.5 min period was recorded. Freezing behavior was defined as the absence of all movements except for respiration. Both context‐ and tone‐related memory tests were recorded by a video recording system. The time of freezing behavior seen in the video was scored by an observer who was blind to group assignment.

### 2.6 Tissue harvest

Mice were deeply anesthetized with isoflurane at 24 h after surgery. The blood was collected, stored overnight at 4°C, and centrifuged at 3000 rpm for 20 min. Serum was collected and stored at −80°C for ELISA of IL‐6 and corticosterone. All mice were perfused with ice‐cold saline before brain harvest. Hippocampus in one hemisphere was dissected out for ELISA of IL‐6. Coronal brain slice of the other hemisphere between Bregma −1 and − 2.5 mm containing the hippocampus was harvested for immunofluorescent staining of Iba‐1. In another experiment, brain slices from Bregma −1.5 to −2.1 mm or − 2.9 to −3.8 were harvested at 3 h after surgery for immunofluorescence staining of c‐Fos in the LHb and VTA.

### 2.7 Quantification of IL‐6 and corticosterone

As we previously described,^13^, ^14^ brain tissues were homogenized on ice in 20 mM Tris–HCL buffer containing protease inhibitors (10 mg/mL aprotinin, 5 mg/mL pepstatin, 5 mg/mL leupeptin and 1 mM phenylmethanesulfonylfluoride). The solutions were centrifuged at 13,000 rpm for 20 min at 4°C. The supernatant was saved and the BCA protein assay of the supernatant was performed for each sample. ELISA kit for measuring IL‐6 (M6000B, R&D Systems) was used to quantify the contents of this cytokine in the samples according to the manufacturer's instructions. The quantity of IL‐6 in each sample was standardized to the protein contents. The content of IL‐6 in the serum was measured in the same way. ELISA kit for measuring corticosterone (Ab108821, Abcam) was used to quantify the amount of corticosterone in the serum.

### 2.8 Immunofluorescent staining

Brains were fixed in 4% paraformaldehyde for 24 h at 4°C and then transferred to 30% sucrose overnight at 4°C before being frozen in optimal cutting temperature compound. Coronal 20‐μm‐thick sections were cut sequentially by using a cryostat and mounted to microscope slides. After being washed in Tris‐buffered saline (TBS), the sections were blocked in 1% bovine serum albumin (BSA) plus 10% donkey serum in TBS with 0.5% Triton‐X100 for 2 h in the humidifying box at room temperature and then incubated with the primary antibodies: rabbit monoclonal anti‐Iba‐1 antibody (1:200, Reagent, catalog number: LEE6003) and rabbit polyclonal anti‐c‐Fos antibody (1:1000, Abcam, catalog number: ab190289), at 4°C overnight. Sections were washed with TBS, and incubated with the secondary antibody donkey anti‐rabbit IgG antibody conjugated with Alexa Fluor 488 (1:200, Invitrogen, catalog number: 2156521) at room temperature in the dark for 1 h. Cell nuclei were stained by Hoechst 33342 (1:1000, Thermo Scientific, catalog number: 62249) at room temperature for 5 min in the dark. Images were acquired with a confocal microscope (Zeiss 700) and the quantification of Iba‐1 was performed as described previously.^13^, ^14^ Briefly, the whole dentate gyrus (DG) region that was covered by 2 to 3 non‐overlapping fields from each of six sequential hippocampus sections of one mouse was imaged. The number of pixels per image with intensity above a predetermined threshold level was considered as a positively stained area for an interested marker, quantified using the Image‐pro plus 6.0, and presented as percentage of positive area in the total area. Six sections per mouse were analyzed. The results of the six sections were averaged to reflect the expression level of a protein in the mouse. All quantitative analyses were performed in a blinded manner.

### 2.9 Statistics and data presentation

No mice or data points were excluded from analysis. All data in normal distribution were presented as mean ± SD with the presentation of data of each individual animal in the bar graphs. Data in non‐normal distribution were presented as median ± interquartile range with the presentation of data of each individual animal in the bar graphs. The normal distribution of the data was accessed by Shapiro–Wilk test. Results of normal distribution were analyzed by using Student's *t* test or one‐way analysis of variance followed with Tukey test and the data of non‐normal distribution were analyzed by using rank sum test or Kruskal–Wallis test followed with Dunn's test as appropriate. Significant differences were accepted at a *p* < 0.05 based on two‐tailed hypothesis testing.

## 3 RESULTS

### 3.1 Living with familiar observers reduced anxiety and dysfunction of learning and memory of mice with surgery

**FIGURE 1:**
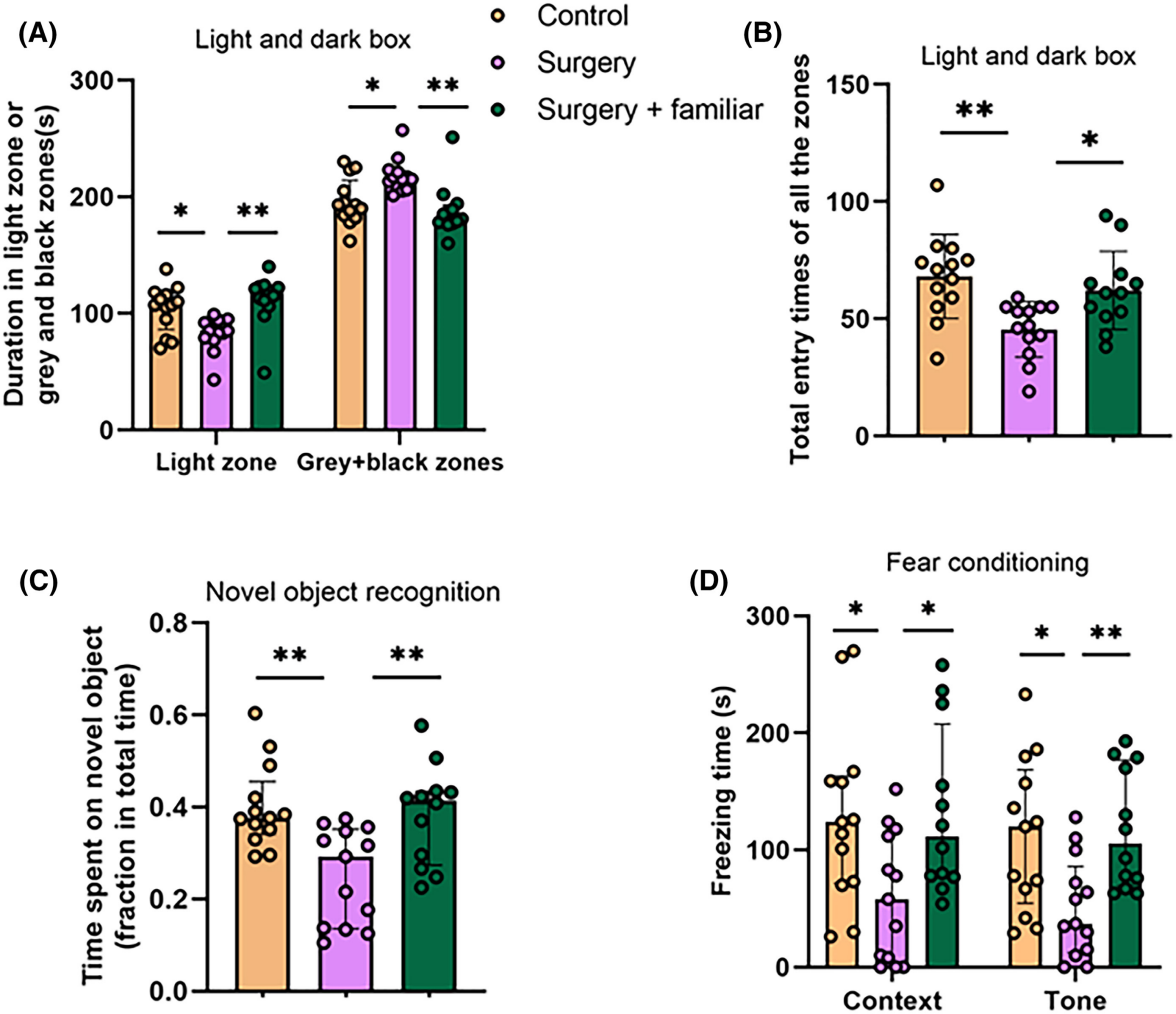
Living with familiar observers attenuated anxiety and the dysfunction of learning and memory after surgery in young adult mice. Familiar observers lived with the mice with surgery for 2 weeks before the surgery and then after the surgery. (A) Duration in various zones of the light and dark box. (B) Total number of entries into various zones of the light and dark box. (C) Performance in novel object recognition test. (D) Performance in fear conditioning test. Results are mean ± SD with the presentation of the value of individual animals (panel B, *n* = 12–13) or median ± interquartile range with the presentation of the value of individual animals (panels A, C, and D, *n* = 12–13). *p < 0.05, **p < 0.001.

Young adult male CD‐1 mice with surgery spent less time in the light zone and more time in the gray and black zones than control mice in the light and dark box test. Also, surgery mice had fewer entries to various zones in this test. These effects were attenuated by living with familiar observers (Figure 1A,B). These results suggest that surgery mice have an increased anxiety, which is attenuated by living with familiar observers.

Mice with surgery spent less time on novel object than control mice in novel object recognition test. This reduction was attenuated by living with familiar cage‐mates (Figure 1C). Similarly, mice with surgery had decreased context‐ and tone‐related freezing behavior. This decrease was attenuated by living with familiar cage‐mates (Figure 1D). These results suggest that surgery induces learning and memory dysfunction and that living with familiar non‐surgery cage‐mates attenuates this impairment.

**FIGURE 2:**
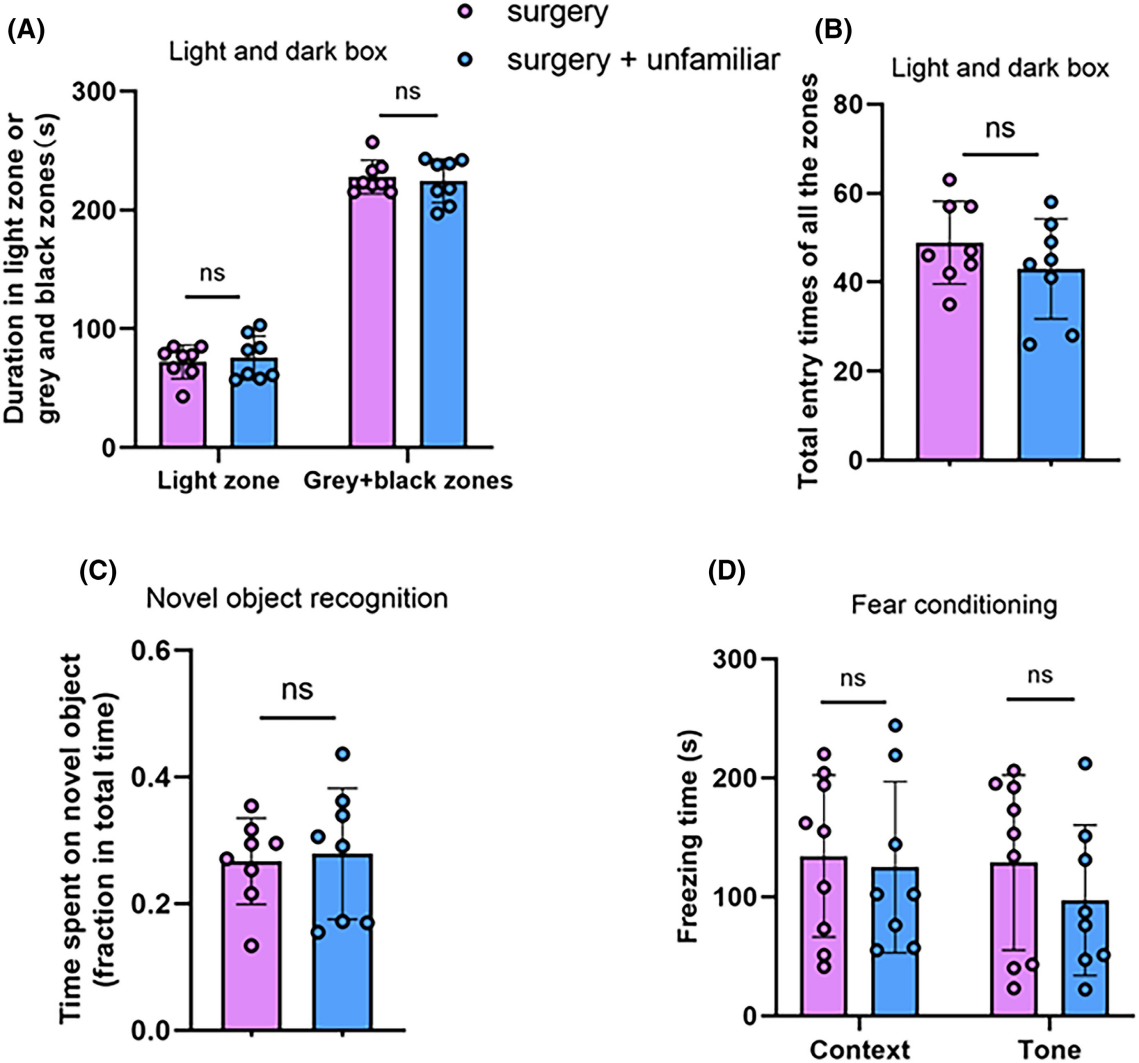
Living with unfamiliar observers did not affect anxiety and the dysfunction of learning and memory after surgery in young adult mice. Unfamiliar observers did not live with the mice with surgery before the surgery but lived with surgery mice after the surgery. (A) Duration in various zones of the light and dark box. (B) Total number of entries into various zones of the light and dark box. (C) Performance in novel object recognition test. (D) Performance in fear conditioning test. Results are mean ± SD with the presentation of the value of individual animals (*n* = 8–9). ns: not significant.

Unlike the situation with familiar cage‐mates, living with unfamiliar non‐surgery cage‐mates after the surgery did not affect the performance of mice with surgery in the light and dark box, novel object recognition, and fear conditioning tests (Figure 2). These results suggest that living with unfamiliar non‐surgery cage‐mates does not reduce anxiety and dysfunction of learning and memory of mice with surgery.

**FIGURE 3:**
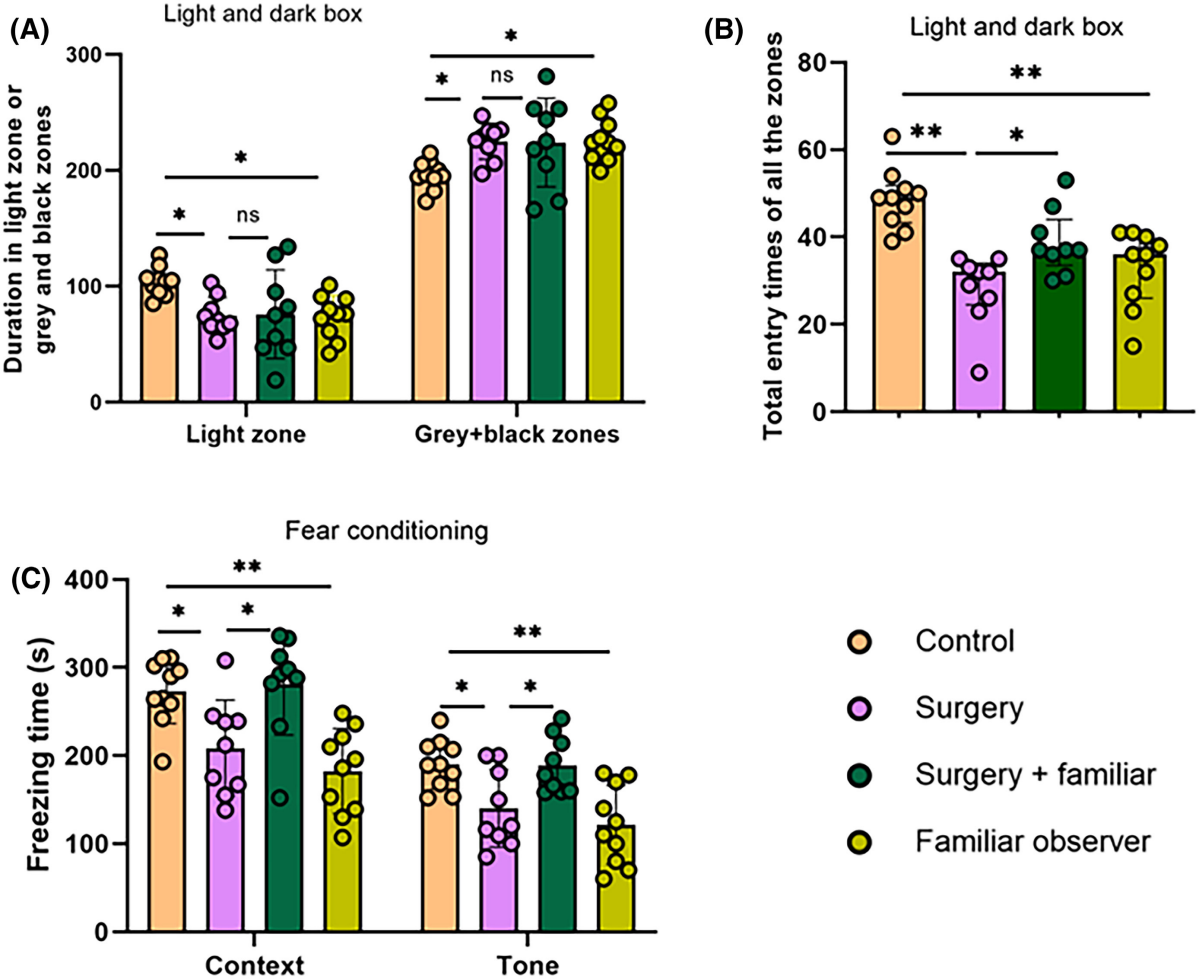
Living with familiar observers attenuated anxiety and the dysfunction of learning and memory after surgery in old mice. Familiar observers lived with the mice with surgery for 2 weeks before the surgery and then after the surgery. (A) Duration in various zones of the light and dark box. (B) Total number of entries into various zones of the light and dark box. (C) Performance in fear conditioning test. Results are mean ± SD with the presentation of the value of individual animals (panels A and C, *n* = 9–10) or median ± interquartile range with the presentation of the value of individual animals (panel B, *n* = 9–10). *p < 0.05, **p < 0.001, ns: not significant.

Similarly, old C57BL/6 male mice with surgery spent less time in the light zone and more time in the gray plus black zones than control mice. The total entry times into all zones were decreased in mice with surgery. However, these effects were not significantly reduced by living with familiar observers, although the time spent in the different zones was not different between control mice and surgery mice living with familiar cage‐mates (Figure 3A,B). These results suggest that surgery also induces anxiety in old mice and that living with familiar cage‐mates may partially reduce this anxiety. Surgery reduced the context‐ and tone‐related freezing behavior and this reduction was attenuated by living with familiar cage‐mates (Figure 3C), suggesting that living with familiar non‐surgery cage‐mates after surgery reduces learning and memory impairment. Interestingly, similar to the situation in young adult mice, familiar non‐surgery cage‐mates of surgery mice spent less time in the light zone and more time in the gray and black zones than control mice. These non‐surgery mice also had less context‐ and tone‐related freezing behavior than control mice (Figure 3). These results suggest that familiar cage‐mates living with surgery mice have increased anxiety and impaired learning and memory.

### 3.2 Living with familiar observers reduced neuroinflammation in mice with surgery

**FIGURE 4:**
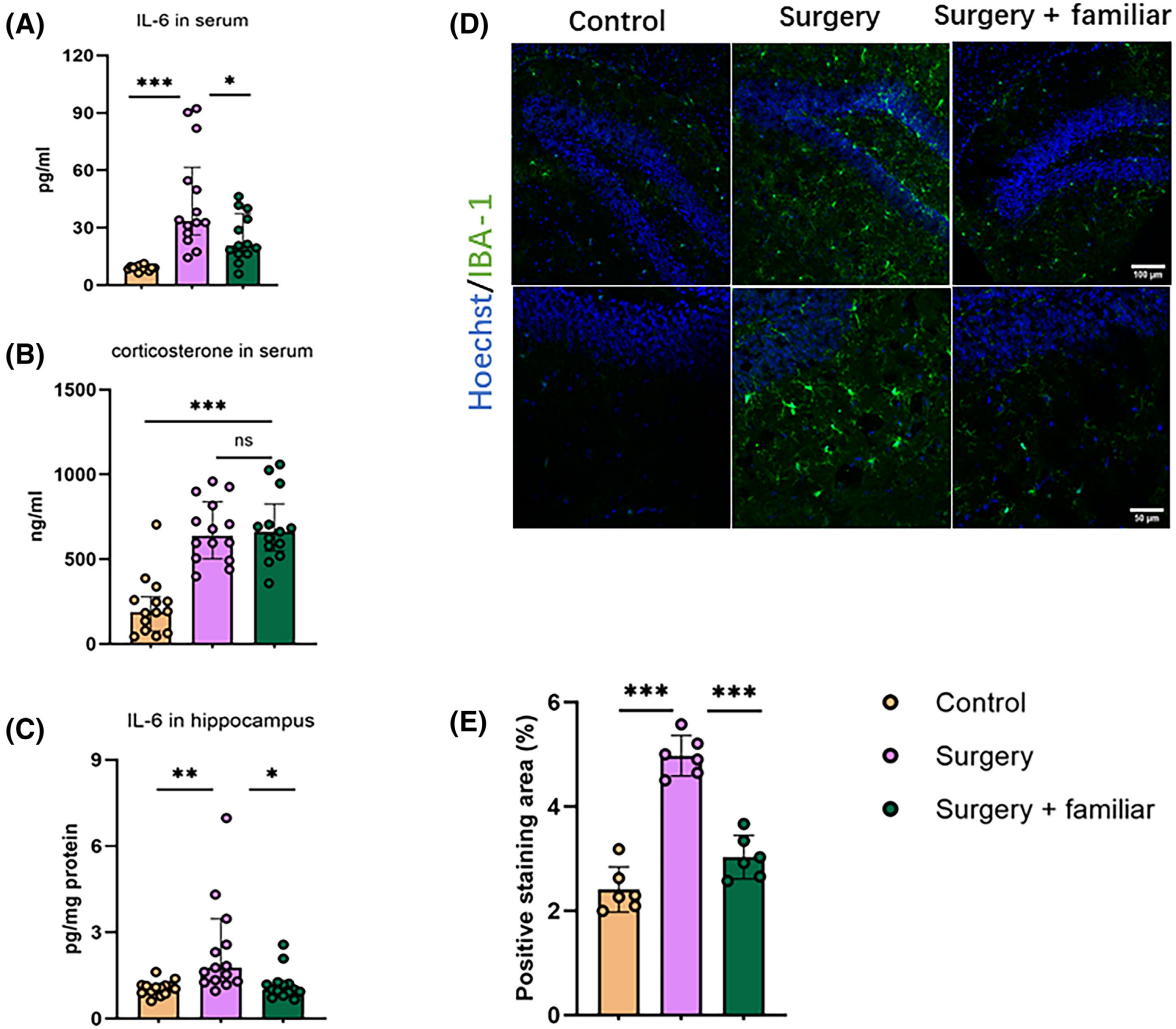
Living with familiar observers attenuated inflammatory responses after surgery in young adult mice. Familiar observers lived with the mice with surgery for 2 weeks before the surgery and then after the surgery. (A) IL‐6 concentrations in the blood. (B) Corticosterone concentrations in the blood. (C) IL‐6 in the hippocampus. (D) Representative images of Iba‐1 staining in the dentate gyrus of the hippocampus. Scale bar = 100 μm in the top panel, =50 μm in the bottom panel. (E) Quantitative results of Iba‐1 staining in the dentate gyrus of the hippocampus. Results are mean ± SD with the presentation of the value of individual animals (panel E, =6) or median ± interquartile range with the presentation of the value of individual animals (panels A to C, *n* = 13–15). *p < 0.05, **p < 0.001, ***p < 0.0001, ns, not significant.

Surgery increased IL‐6 and corticosterone in the blood of young adult mice. The IL‐6 increase was reduced by living with familiar cage‐mates (Figure 4A,B). Surgery also increased IL‐6‐ and Iba‐1‐positive staining in the hippocampus. These increases were attenuated by living with familiar non‐surgery cage‐mates after surgery (Figure 4C,D). These results suggest that surgery induces a general inflammatory response and neuroinflammation, which are attenuated by living with familiar cage‐mates.

### 3.3 The activation of the LHb‐VTA neural circuitry was inhibited by living with familiar observers or bupivacaine infiltration to the surgical wound

**FIGURE 5:**
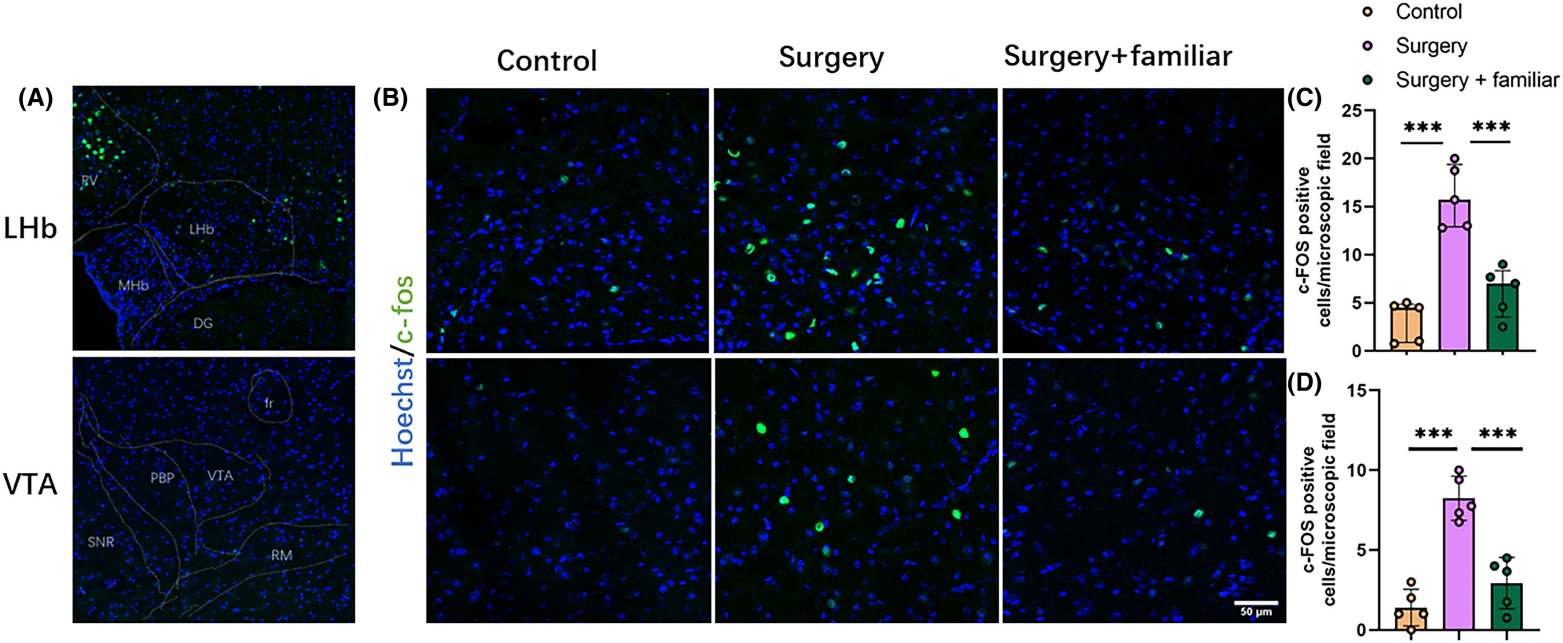
Living with familiar observers reduced the number of c‐Fos‐positive cells in the LHb‐VTA after surgery in young adult mice. Familiar observers lived with the mice with surgery for 2 weeks before the surgery and then after the surgery. (A) Representative images of brain sections with the brain regions identified. (B) Representative images of c‐Fos staining in the LHb and VTA. Scale bar = 50 μm. (C) Quantitative results of the number of c‐Fos‐positive cells in the LHb. (E) Quantitative results of the number of c‐Fos‐positive cells in the VTA. Results are mean ± SD with the presentation of the value of individual animals (panel D, *n* = 5) or median ± interquartile range with the presentation of the value of individual animals (panel C, *n* = 5). ***p < 0.0001. DG, dentate gyrus; Fr, fasciculus retroflexus; MHb, medial habenular nucleus; PBP, parabrachial pigmented area; PV, praventricular thalamic nucleus; RM, retromanmmilary nucleus; SNR, substantia nigra reticulate.

**FIGURE 6:**
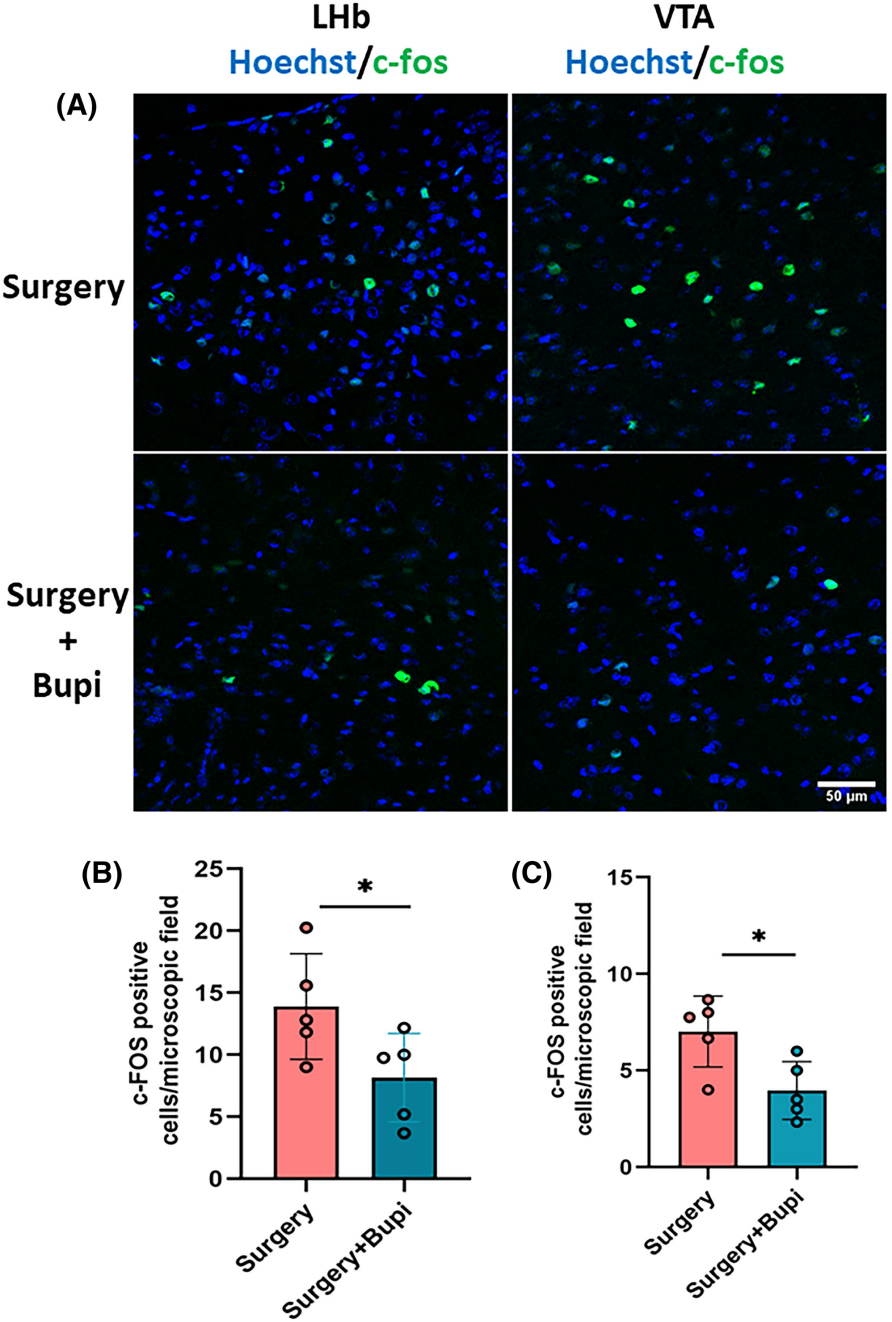
Infiltration with bupivacaine reduced the number of c‐Fos‐positive cells in the LHb‐VTA after surgery in young adult mice. Mice with surgery were infiltrated with or without bupivacaine locally to the surgical wound. (A) Representative images of c‐Fos staining in the LHb and VTA. Scale bar = 50 μm. (B) Quantitative results of the number of c‐Fos‐positive cells in the LHb. (C) Quantitative results of the number of c‐Fos‐positive cells in the VTA. Results are mean ± SD with the presentation of the value of individual animals (*n* = 5). * *p* < 0.05. Bupi, bupivacaine.

Surgery increased c‐Fos‐positive cells in the LHb and VTA. This increase was inhibited by living with familiar cage‐mates (Figure 5). The number of c‐Fos‐positive cells in the LHb and VTA of mice with surgery was also reduced by bupivacaine infiltration to the surgical wound (Figure 6). These results suggest that surgery activates LHb and VTA, which is inhibited by living with familiar cage‐mates and local anesthetic infiltration to the wound.

## 4 DISCUSSION

Consistent with our previous study,^7^ mice with surgery have increased anxiety and living with familiar cage‐mates reduces this anxiety. Our current study extends the previous finding by showing that living with familiar non‐surgery cage‐mates reduces learning and memory impairment in young and old mice with surgery. Excessive anxiety is unhealthy and can impair learning and memory.^5^, ^6^ It is possible that the reduced learning and memory dysfunction of surgery mice living with familiar cage‐mates may be due to the attenuated anxiety in these surgery mice.

Mice with surgery had increased IL‐6 in the blood, suggesting a systemic inflammatory response as shown in our previous study.^15^ These mice also had increased corticosteroid, suggesting a stress response. Surgery also increased the concentrations of IL‐6 and the level of Iba‐1 in the hippocampus, suggesting that surgery induces neuroinflammation. These effects, except for the increase of corticosteroid in the blood, were inhibited by living with familiar observers. These results suggest that living with familiar observers reduces systemic inflammation and neuroinflammation in mice with surgery. These results are consistent with the functional results that living with familiar observers attenuates learning and memory dysfunction in mice with surgery and the consideration that neuroinflammation is an underlying neuropathological process for POCD.^8^, ^9^

We have shown that familiar observers living with mice with surgery have increased anxiety^7^ and that non‐surgery mice living with surgery mice can develop learning and memory dysfunction in young adult mice.^16^ Consistent with these results, familiar non‐surgery old mice living with old mice with surgery also had increased anxiety and dysfunction of learning and memory. These results suggest that increased anxiety is linked to learning and memory dysfunction during the perioperative period. These results also indicate the importance of reducing anxiety in both mice with surgery and their non‐surgery cage‐mates to improve their health.

The neural circuitry LHb‐VTA is shown to play an important role in POCD in our previous study.^10^ Consistent with that finding, mice with surgery had a higher number of cells that were positive for c‐Fos in the LHb and VTA. This increase was inhibited by living with familiar observers. These results suggest that inhibiting the activation of LHb and VTA is a mechanism for living with familiar observers to attenuate the learning and memory dysfunction of mice with surgery, providing further evidence for the role of the neural circuitry LHb‐VTA in the development of POCD. Interestingly, infiltration of the surgical wound with bupivacaine reduced the activation of LHb and VTA and attenuates learning and memory dysfunction after surgery.^11^ These results suggest that neurotransmission from peripheral stimulation, likely the pain, is important for activating the LHb‐VTA circuitry.

Our findings may have significant implications. Living with familiar observers but not with unfamiliar observers reduces learning and memory dysfunction of mice with surgery, implying that living with family members or friends after surgery may reduce POCD. Currently, there are no available data to suggest this finding in humans. Future clinical studies may test whether our findings in animals are applicable to humans. In addition, future studies can determine the role of pain in the development of POCD. It is important to note that human relationships are very sophisticated. The family living patterns are affected by multiple factors including culture and financial conditions, which also affects whether the patients with surgery will have the company of their family member(s).

Our study has limitations. First, we used male animals in our study. It is not clear whether our findings shown here are applicable in female animals. However, there is no evidence showing that the incidence of POCD is different between males and females in humans.^1^, ^2^ Second, aging is a risk factor for POCD observed a few weeks or months later. Our results have shown that living with familiar observers reduced learning and memory impairment of surgery mice and that familiar observers develop learning and memory dysfunction in the old mouse experiments. These results suggest similar effects to those of young adult mice after the interaction between familiar observers and surgery mice. However, the molecular and neural mechanisms for these effects in old mice are not known and will be investigated in a future study.

## 5 CONCLUSION

Our study has shown that living with familiar observers attenuates POCD and neuroinflammation in mice with surgery. Reducing the activation of LHb‐VTA neural circuitry may be a mechanism for these effects.

## AUTHOR CONTRIBUTIONS

ZZ conceived the project. QJ and ZZ designed the studies. QJ and MG performed the experiments. QJ did initial data analysis and drafted methods section. ZZ performed the final analysis of the data and wrote the manuscript.

## FUNDING INFORMATION

This study was supported by grants (RF1 AG061047 and R01 HD089999 to Z Zuo) from the National Institutes of Health, Bethesda, MD, USA and the Robert M. Epstein Professorship endowment (to Z Zuo), University of Virginia, Charlottesville, VA, USA.

## CONFLICT OF INTEREST STATEMENT

None.

## ETHICAL APPROVAL AND CONSENT TO PARTICIPATE

All animal procedures were approved by our institute animal care and use committee.

## Data Availability

The data are available upon a reasonable request.

## References

[1] Monk TG , Weldon BC , Garvan CW , et al. Predictors of cognitive dysfunction after major noncardiac surgery. Anesthesiology. 2008;108:18‐30. 18156878 10.1097/01.anes.0000296071.19434.1e

[2] Li Y , Chen D , Wang H , et al. Intravenous versus volatile anesthetic effects on postoperative cognition in elderly patients undergoing laparoscopic abdominal surgery. Anesthesiology. 2021;134:381‐394. doi:10.1097/ALN.0000000000003680 33439974

[3] Steinmetz J , Christensen KB , Lund T , Lohse N , Rasmussen LS . Long‐term consequences of postoperative cognitive dysfunction. Anesthesiology. 2009;110:548‐555. 19225398 10.1097/ALN.0b013e318195b569

[4] Mulugeta H , Ayana M , Sintayehu M , Dessie G , Zewdu T . Preoperative anxiety and associated factors among adult surgical patients in Debre Markos and Felege Hiwot referral hospitals, Northwest Ethiopia. BMC Anesthesiol. 2018;18:155. doi:10.1186/s12871-018-0619-0 30376809 PMC6208029

[5] Zuo Z . Have we forgotten something when caring for patients for surgery? Front Med (Lausanne). 2022;9:952893. doi:10.3389/fmed.2022.952893 35966850 PMC9366056

[6] Hauck A , Michael T , Ferreira de Sa DS . Fear learning and generalization during pandemic fear: how COVID‐19‐related anxiety affects classical fear conditioning with traumatic film clips. J Psychiatr Res. 2022;155:90‐99. doi:10.1016/j.jpsychires.2022.07.068 35998471 PMC9365308

[7] Zeng Q , Shan W , Zhang H , Yang J , Zuo Z . Paraventricular thalamic nucleus plays a critical role in consolation and anxious behaviors of familiar observers exposed to surgery mice. Theranostics. 2021;11:3813‐3829. doi:10.7150/thno.45690 33664863 PMC7914349

[8] Zheng B , Lai R , Li J , Zuo Z . Critical role of P2X7 receptors in the neuroinflammation and cognitive dysfunction after surgery. Brain Behav Immun. 2017;61:365‐374. doi:10.1016/j.bbi.2017.01.005 28089560 PMC5316360

[9] Cao L , Li L , Lin D , Zuo Z . Isoflurane induces learning impairment that is mediated by interleukin 1beta in rodents. PloS One. 2012;7:e51431. doi:10.1371/journal.pone.0051431 23251531 PMC3520904

[10] Xin J , Shan W , Li J , Yu H , Zuo Z . Activation of the lateral habenula‐ventral tegmental area neural circuit contributes to postoperative cognitive dysfunction in mice. Adv Sci (Weinh). 2022;9:e2202228. doi:10.1002/advs.202202228 35616407 PMC9353455

[11] Chi H , Kawano T , Tamura T , et al. Postoperative pain impairs subsequent performance on a spatial memory task via effects on N‐methyl‐D‐aspartate receptor in aged rats. Life Sci. 2013;93:986‐993. doi:10.1016/j.lfs.2013.10.028 24211778

[12] Min J , Lai Z , Wang H , Zuo Z . Preoperative environment enrichment preserved neuroligin 1 expression possibly via epigenetic regulation to reduce postoperative cognitive dysfunction in mice. CNS Neurosci Ther. 2021;28:619‐629. doi:10.1111/cns.13777 34882968 PMC8928916

[13] Lai Z , Shan W , Li J , Min J , Zeng X , Zuo Z . Appropriate exercise level attenuates gut dysbiosis and valeric acid increase to improve neuroplasticity and cognitive function after surgery in mice. Mol Psychiatry. 2021;26:7167‐7187. doi:10.1038/s41380-021-01291-y 34663905 PMC8873004

[14] Mei B , Li J , Zuo Z . Dexmedetomidine attenuates sepsis‐associated inflammation and encephalopathy via central alpha2A adrenoceptor. Brain Behav Immun. 2021;91:296‐314. doi:10.1016/j.bbi.2020.10.008 33039659 PMC7749843

[15] Zhang J , Tan H , Jiang W , Zuo Z . The choice of general anesthetics may not affect neuroinflammation and impairment of learning and memory after surgery in elderly rats. J Neuroimmune Pharmacol. 2015;10:179‐189. 25649847 10.1007/s11481-014-9580-y

[16] Zheng Y , Zuo Z . Learning and memory dysfunction of non‐surgery cage‐mates of mice with surgery. Stress. 2020;23:474‐480. doi:10.1080/10253890.2019.1702641 31820673 PMC7295679

